# Early *BCR::ABL1* Reduction as a Predictor of Deep Molecular Response in Pediatric Chronic-Phase Chronic Myeloid Leukemia

**DOI:** 10.3390/cancers17243994

**Published:** 2025-12-15

**Authors:** Xingchen Wang, Wenbin An, Chenmeng Liu, Bang Zhang, Yunlong Chen, Yang Wan, Xiaolan Li, Lipeng Liu, Fang Liu, Li Zhang, Yao Zou, Xiaojuan Chen, Yumei Chen, Ye Guo, Tianyuan Hu, Yingchi Zhang, Xiaofan Zhu, Wenyu Yang

**Affiliations:** 1 State Key Laboratory of Experimental Hematology, National Clinical Research Center for Blood Diseases, Haihe Laboratory of Cell Ecosystem, Institute of Hematology & Blood Diseases Hospital, Chinese Academy of Medical Sciences & Peking Union Medical College, Tianjin 300020, China; wangxingchen@ihcams.ac.cn (X.W.); anwenbin@ihcams.ac.cn (W.A.);; 2 Tianjin Institute of Health Science, Tianjin 301600, China

**Keywords:** pediatric chronic myeloid leukemia, *BCR::ABL1*, deep molecular remission, TKI

## Abstract

**Background**: Tyrosine kinase inhibitors (TKIs) have transformed the prognosis of chronic myeloid leukemia (CML), but pediatric patients face unique challenges due to prolonged exposure. Early molecular response (EMR, *BCR::ABL1* ≤ 10% at 3 months) is a recognized predictor of favorable outcomes in adults and has been correlated with improved responses in children. However, its relationship with achieving deep molecular remission (DMR, *BCR::ABL1* ≤ 0.01%) in pediatric CML remains unclear. **Methods**: We performed a single-center, retrospective analysis of 103 pediatric patients with chronic-phase CML treated with frontline TKIs. Among them, 88 were evaluable for molecular response. *BCR::ABL1* transcript levels were quantified by real-time quantitative PCR on the International Scale, and molecular responses were assessed. Associations between early molecular dynamics and long-term outcomes were evaluated using Kaplan–Meier and cumulative incidence analyses. **Results**: At 3 months, 64.8% achieved EMR. Early responders had significantly higher MMR rates at 12 months (80.8% vs. 5.6%; *p* = 0.00018) and DMR at 24 months (70.4% vs. 42.2%; *p* = 0.029). The ≥0.45-log reduction in *BCR::ABL1* transcripts at 3 months predicted shorter times to MMR (median 11 vs. 29 months) and DMR (18 vs. 50 months), as well as higher overall MMR (*p* = 0.011) and DMR (*p* = 0.014) incidences. Bone marrow fibrosis correlated with inferior molecular outcomes (*p* = 0.017 for MMR). **Conclusions**: Early *BCR::ABL1* decline kinetics independently predict molecular depth in pediatric CML. Quantitative early transcript reduction may guide risk-adapted management and optimize long-term TKI strategies in children.

## Simple Summary

Children with chronic myeloid leukemia require long-term treatment with tyrosine kinase inhibitors, which can lead to unique challenges compared with adults. Identifying early markers that predict long-term treatment success could help doctors tailor therapy and reduce unnecessary exposure. In this study, we analyzed children and adolescents with chronic myeloid leukemia who received first-line tyrosine kinase inhibitor therapy. We measured the rate at which the leukemia marker *BCR::ABL1* decreased within the first three months and compared it with the long-term treatment outcome. We found that patients whose *BCR::ABL1* levels dropped rapidly in the early stage of treatment were more likely to reach deeper and faster remission. Patients with bone marrow fibrosis showed poorer responses. These findings highlight that early reduction in leukemia markers can serve as a useful indicator for evaluating long-term prognosis, and it can also assist in formulating individualized treatment plans for children.

## 1. Introduction

Pediatric chronic myeloid leukemia (CML) is a rare hematologic malignancy that typically presents with a more aggressive clinical phenotype at diagnosis compared to adult cases. Its global annual incidence is estimated at 0.6–1.2 per million, with incidence rates increasing with age [1,2,3,4]. Despite these more pronounced clinical manifestations at presentation, both prospective and retrospective pediatric studies have demonstrated that, with tyrosine kinase inhibitors (TKIs) therapy, children achieve long-term outcomes comparable to those of adults. The introduction of TKIs therapy has markedly improved the prognosis of CML, allowing adult patients in the chronic phase to achieve near-normal life expectancies [4,5]. Nevertheless, children undergoing TKI treatment face distinctive challenges due to the potential impacts of long-term therapy on growth and development [5,6].

Disease monitoring in CML relies on serial quantification of *BCR::ABL1* transcript levels [7]. Key molecular milestones are evaluated at standardized intervals (3, 6, and 12 months) in accordance with international guidelines, which are primarily derived from adult studies [8]. Attaining these milestones carries significant prognostic implications. For instance, the European Leukemia Net (ELN) 2020 guidelines define *BCR::ABL1* > 10% at 3 months as treatment failure, highlighting the critical importance of early response [7].

A rapid reduction in *BCR::ABL1* transcripts following TKI initiation is a well-documented predictor of favorable long-term outcomes. In adults, *BCR::ABL1* ≤ 10% at 3 months is associated with improved progression-free and overall survival, while slower responses correlate with higher risks of treatment failure [9,10,11]. Pediatric studies corroborate these observations: children who achieve *BCR::ABL1* ≤ 10% at 3 months are significantly more likely to attain complete cytogenetic response and major molecular response (MMR) by 12 months, and exhibit superior progression-free survival compared to those above this threshold. A French cohort study confirmed that early molecular response strongly predicts clinical outcomes in pediatric CML [12]. Recent investigations have further refined prognostic assessment by evaluating quantitative kinetic parameters, such as *BCR::ABL1* halving time, to predict deep molecular responses in adult patients [13,14,15,16,17,18].

A key therapeutic goal in CML is achieving sustained deep molecular response (DMR, *BCR::ABL1* ≤ 0.01%), which may allow for treatment discontinuation aimed at treatment-free remission (TFR). Sustained DMR is a prerequisite for TKI cessation in both adult guidelines and emerging pediatric recommendations [7,8,19,20]. Thus, achieving DMR as early as possible is particularly desirable in children to minimize cumulative TKI exposure. Given the prognostic value of early molecular response (EMR) and the ultimate objective of TFR, there is a growing need to elucidate how early *BCR::ABL1* kinetics influence long-term molecular outcomes in pediatric CML. Although studies in adults have shown that rapid early transcript reduction is associated with DMR, pediatric-specific data remain scarce and have largely focused on the dichotomous 3-month EMR threshold (*BCR::ABL1* ≤ 10% versus >10%) in relation to cytogenetic or MMR and event-free survival (EFS), rather than the depth and timing of DMR [12,13,21,22,23].

In this study, we analyzed a single-center cohort of pediatric patients with chronic-phase CML receiving TKI therapy to assess whether EMR indicators can predict the attainment and timing of both MMR and DMR. Specifically, we quantified early *BCR::ABL1* decline using a 3-month log-reduction metric in addition to the conventional 10% cut-off, and examined how these early kinetics relate to subsequent molecular depth. We also investigated additional factors associated with these molecular responses, aiming to provide insights that could guide clinical management in pediatric CML.

## 2. Materials and Methods

### 2.1. Patients

We conducted a single-center, retrospective cohort study of children and adolescents who aged <18 years who were diagnosed with Philadelphia chromosome-positive CML in chronic phase (CML-CP) between November 2002 and February 2025. Patients were excluded if they initiated TKI therapy more than 6 months after diagnosis or lacked essential longitudinal molecular or treatment response data. The sample size was determined by the number of consecutive eligible children with CML-CP who initiated TKI therapy at our center during the study period; no formal a priori sample size or power calculation was performed. The study was approved by the Ethics Committee of Blood Diseases Hospital, Chinese Academy of Medical Sciences, and was conducted in accordance with the principles of the Good Clinical Practice guidelines and the Declaration of Helsinki.

### 2.2. Treatment

The choice of initial TKI was determined through shared decision-making between physicians and parents of the patients, considering disease risk, treatment objectives, safety–efficacy profiles of available agents, and financial considerations. Initial dosing regimens were as follows: imatinib at 260–340 mg/m^2^ once daily (maximum 400 mg/d), dasatinib at 60 mg/m^2^ once daily (maximum 100 mg/d), and nilotinib at 230 mg/m^2^ (maximum 400 mg) twice daily. Subsequent adjustments to the TKI type or dosage were made based on treatment response, tolerability, and logistical factors, in alignment with the latest ELN recommendations [7,24,25,26].

### 2.3. Molecular Analysis and Assessment of Treatment Response

For the quantification of *BCR::ABL1*, our institution conducted qualitative methods from 2003 to 2010, with a minimal target sensitivity of 10−4 [27]. Since 2010, we have switched to quantitative methods with a minimal target sensitivity of 10^−5^ [28]. In 2012, through the CF confirmation project led by Peking University People’s Hospital, we determined our CF value to be 0.9. Since 2013, international standard values have been applied to represent the quantification of *BCR::ABL1* [29]. Diagnosis, monitoring, and definitions of cytogenetic and molecular responses adhered to the ELN recommendations [7,24,25,26]. Cytogenetic analysis was performed on bone marrow samples using G-banding. Additional chromosomal abnormalities (ACAs) were categorized according to established criteria [7]. The EUTOS long-term survival (ELTS) score was calculated at diagnosis as previously described [30]. Bone marrow fibrosis (BMF) was assessed on reticulin-stained bone marrow biopsy sections and graded (MF-0 to MF-3) according to the European Consensus system [31].

Complete hematologic response (CHR) was defined as follows: white blood cell count (WBC) < 10 × 10^9^/L, platelet count < 450 × 10^9^/L, peripheral blood basophils < 5%, a normal differential count, resolution of all disease-associated symptoms, and a non-palpable spleen. Complete cytogenetic response (CCyR) was defined by 0% Ph+ metaphases in the bone marrow. MMR was defined as *BCR::ABL1* ≤ 0.1%. DMR was defined as *BCR::ABL1* ≤ 0.01%. Overall survival (OS) was measured from TKI initiation until death or the last follow-up. Failure-free survival (FFS) was defined as the time from TKI initiation until failure to meet ELN 2020 response milestones, progression to accelerated phase (AP) or blast phase (BP) [7], death or the last follow-up. Progression-Free Survival (PFS) was calculated from TKI initiation until transformation to AP or BP, death, or the last follow-up. Event-free survival (EFS) was defined as the time from TKI initiation until loss of CHR, CCyR, or MMR, ELN-defined treatment failure, emergence of *BCR::ABL1* mutations, progression to AP/BP, death from any cause or last follow-up. The cut-off date for follow-up was 1 July 2025.

### 2.4. Statistical Analysis

Continuous variables were summarized as median (range). Categorical variables were presented by frequency (percentage). Cumulative incidences of CCyR, MMR, MR4 and MR5 was calculated using the Fine-Gray test considering competing events defined as hematopoietic stem cell transplantation, permanent TKI discontinuations for any reason, or death prior to achieving therapeutic response. Patients who had neither achieved the respective molecular endpoint nor experienced a competing event were censored at the date of their last available molecular assessment. Missing *BCR::ABL1* values at specific time points were not imputed. Hazard ratios (HRs) with 95% confidence intervals (CIs) for key associations were estimated using Cox proportional hazards regression. For MMR and DMR, multivariable Cox models included 3-month *BCR::ABL1* kinetics (*BCR::ABL1* ≤ 10% vs.> 10% or log-reduction ≥ 0.45 vs.< 0.45) together with age at diagnosis, baseline BMF, spleen size, TKI switching and ELTS risk category as covariates. OS, FFS, PFS and EFS and survival rates were calculated by using the Kaplan–Meier method and compared by log-rank test. For all Kaplan–Meier analyses, time was measured from TKI initiation to the first occurrence of the corresponding event as defined above. Patients who remained free of the event of interest were censored at the date of last documented contact, and those lost to follow-up were censored at the date of their last recorded visit. The rate of *BCR::ABL1* decline was evaluated by calculating the log reduction from baseline to 3 months, defined as log (transcript level at diagnosis/transcript level at 3 months). A two-sided *p*-values < 0.05 were considered statistically significant. All analyses were performed using SPSS (version 26.0) and R statistical software (version 4.3.1).

## 3. Results

### 3.1. Patient and Baseline Characteristics

**Figure 1:**
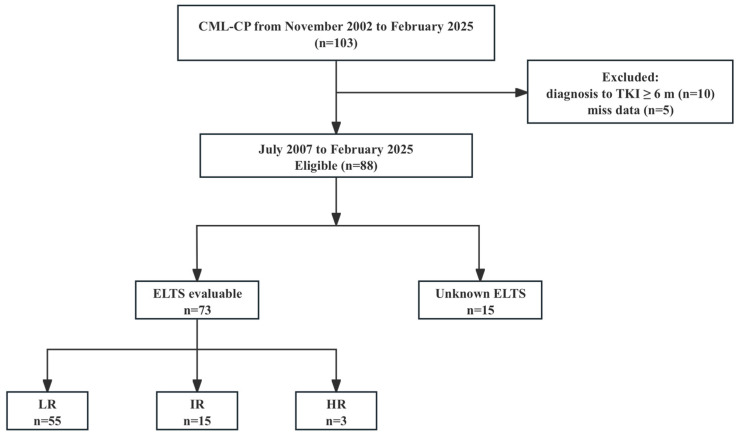
Flow diagram of pediatric CML-CP cohort.

Between November 2002 and February 2025, a total of 103 patients who were younger than 18 years at diagnosis with chronic-phase CML were identified at our institution. After excluding 10 patients who initiated TKI therapy more than 6 months post diagnosis and 5 patients with incomplete essential data, 88 children remained in the final analytic cohort (Figure 1).

The median age at diagnosis was 9.2 years (range, 1.0–17.0), and there was a male predominance (*n* = 64, 72.7%) over girls (*n* = 24, 27.3%). The most common presenting symptoms were fever (*n* = 28, 31.8%), incidental diagnosis in asymptomatic patients (*n* = 25, 28.4%), and fatigue (*n* = 24, 27.3%). Splenomegaly was observed in 61 patients (69.3%), with a median spleen size of 9.0 cm below the left costal margin (range, 1.0–25.3 cm), and 25 (28.4%) had hepatomegaly. At diagnosis, an ELTS risk score was available in 73 patients; 55 (62.5%) were low risk, 15 (17.0%) intermediate risk, and 3 (3.4%) high risk. The baseline demographic and clinical characteristics are summarized in Table 1.

### 3.2. TKI Treatment, Response Milestones, and Long-Term Outcomes

Among the 88 children with CML-CP, the median time from diagnosis to initiation of TKI therapy was 4 days (range, 1–65). First-line treatment was imatinib in 86 (97.7%). During follow-up, 6 children (6.8%) received TKI therapy for less than 1 year, while 64 (72.7%) were treated for more than 2 years. A total of 25 (28.4%) patients switched TKI during treatment, and 4 patients required third-line or beyond (Figure S1, Table S1). Among the 25 patients who switched TKIs, the main reasons for the first switch were resistance (*n* = 12, 48%), suboptimal response (*n* = 6, 24%), intolerance (*n* = 4, 16%), and personal reasons (*n* = 3, 12%). At the last follow-up, 75 (85.2%) remained on TKIs, including imatinib (*n* = 54, 61.4%), dasatinib (*n* = 13, 14.8%), nilotinib (*n* = 2, 2.3%), olverembatinib (*n* = 2, 2.3%), flumatinib (*n* = 1, 1.1%), and ponatinib (*n* = 1, 1.1%). In China, olverembatinib and flumatinib are approved only for adult CML; in this cohort, pediatric use occurred exclusively within clinical trials. Therapy was discontinued in six patients (6.8%) due to self-discontinuation (*n* = 2), following transplantation (*n* = 2), or following physician-guided TFR attempt (*n* = 2).

During the entire follow-up, 20 patients experienced treatment failure, 8 experienced a loss of MMR, 3 developed disease progression, and 7 were lost to follow-up. At 3 months, 80 patients were evaluable for CHR, of whom 76 (95.0%) achieved CHR. Using ELN categories among those evaluable at each landmark, optimal/warning/failure were 68.4%/21.1%/10.5% at 3 months (39/57, 12/57, 6/57), 60.0%/32.0%/8.0% at 6 months (30/50, 16/50, 4/50), and 53.8%/26.9%/19.2% at 12 months (28/52, 14/52, 10/52), respectively (Table 2).

**Figure 2:**
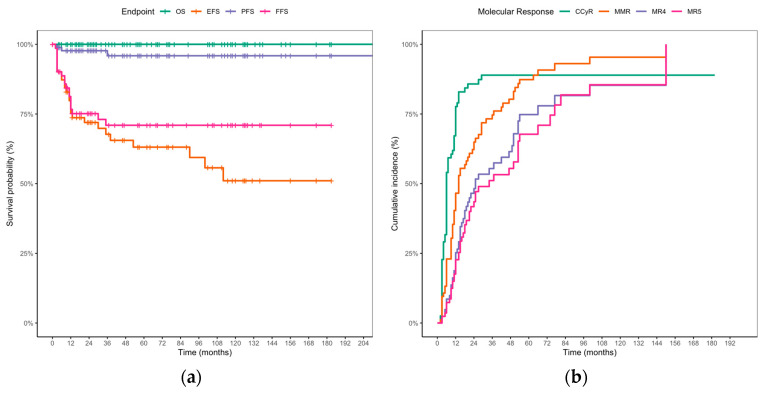
**Survival Endpoints and Cumulative Molecular Responses in Pediatric CML-CP on TKI Therapy:** (**a**) OS, EFS, FFS, and PFS curves; (**b**) cumulative incidence of CCyR, MMR, MR4, and MR5.

With a median follow-up of 56.5 months (4–215), the 5-year OS, PFS, EFS, and FFS were 100% (95% CI, 100–100), 95.9% (95% CI, 91.3–100), 63.1% (95% CI, 51.8–76.9), and 71.0% (95% CI, 60.6–83.1), respectively (Figure 2a). The 5-year FFS was 78.1% (95% CI, 66.4–91.9) in ELTS low-risk patients versus 53.6% (95% CI, 31.9–90.1) in ELTS intermediate/high-risk patients (*p* = 0.095). The corresponding 5-year EFS was 71.3% (95% CI, 57.8–88.0) versus 53.6% (95% CI, 31.9–90.1), respectively (*p* = 0.23) (Figure S2). The 5-year cumulative incidence of CCyR, MMR, MR4, and MR5 was 88.9% (95% CI, 78.7–94.3), 87.3% (95% CI, 76.9–93.1), 74.8% (95% CI, 59.8–84.2), and 67.7% (95% CI, 51.9–78.4), respectively (Figure 2b). The median times to CCyR, MMR, MR4, and MR5 were 6 months (95% CI, 6–11), 12 months (95% CI, 10–15), 20 months (95% CI, 15–47), and 22 months (95% CI, 18–53), respectively.

### 3.3. Prognostic Impact of 3-Month Molecular Response on MMR and DMR

**Figure 3:**
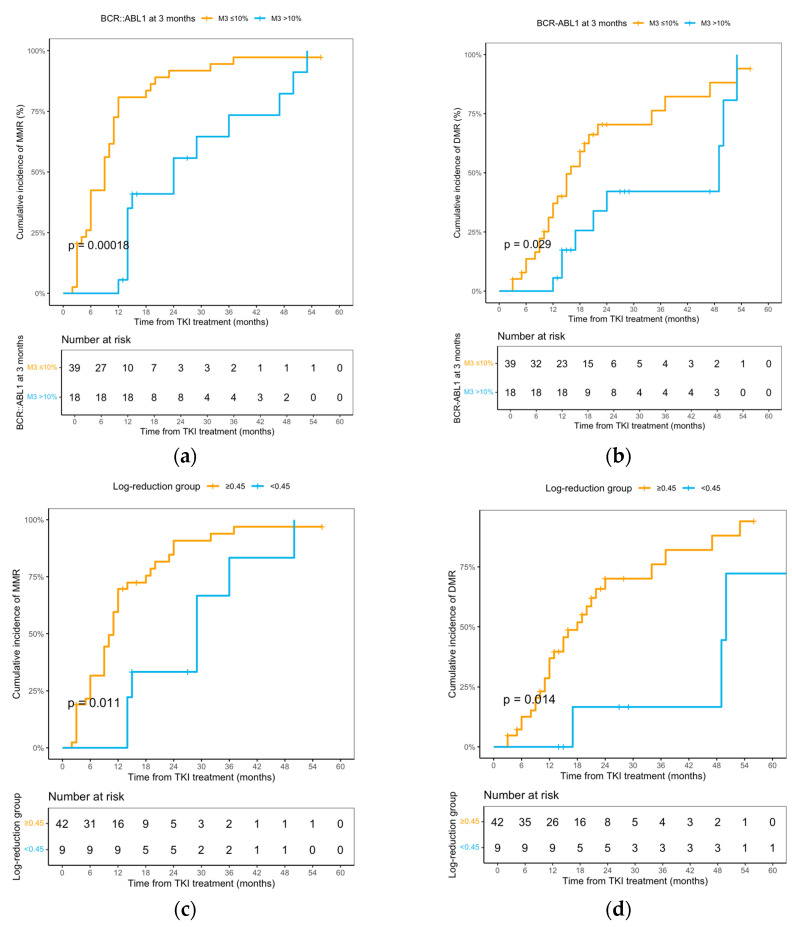
**Cumulative incidence of MMR and DMR stratified by 3-month *BCR::ABL1* transcript dynamics.** (**a**,**b**) Stratification based on absolute transcript levels at 3 months (≤10% vs. >10%); (**c**,**d**) stratification based on log-reduction from baseline to 3 months (≥0.45 vs. <0.45).

Among 88 children with CML-CP, 57 (64.8%) had a 3-month *BCR::ABL1* assessment; the median 3-month *BCR::ABL1* level was 3.55% (range, 2.88–114.24%). Based on a 10% IS cut-off, 39/57 (68.4%) had *BCR::ABL1* ≤ 10% and 18/57 (31.6%) had >10%. In the ≤10% group, 36 patients achieved MMR (median time: 9 months; 95% CI, 6–11) and 27 achieved DMR (median time: 16 months; 95% CI, 12–34). The cumulative incidences of MMR and DMR were significantly higher in the ≤10% group compared with the >10% group (12-month MMR: 80.8% vs. 5.6%; HR, 2.35; 95% CI, 1.21–4.56; *p* = 0.00018; 24-month DMR: 70.4% vs. 42.2%; HR, 1.79; 95% CI, 0.81–3.96; *p* = 0.029; Figure 3a,b). Compared with patients with a 3-month log-reduction ≥ 0.45, those with a log-reduction < 0.45 had significantly poorer EFS (HR, 0.16; 95% CI, 0.05–0.46; *p* = 0.00026) and FFS (HR, 0.09; 95% CI, 0.02–0.34; *p* < 0.0001; Figure S3). In multivariable Cox models adjusting for age, baseline bone marrow fibrosis, spleen size, TKI switching and ELTS risk, 3-month *BCR::ABL1* ≤ 10% was associated a tendency to achieve more MMR and DMR compared with >10%, although these associations did not reach conventional statistical significance (MMR: HR, 2.55; 95% CI, 0.91–7.19; *p* = 0.076; DMR: HR, 5.78; 95% CI, 0.96–34.68; *p* = 0.055).

Of the 61 children who achieved MMR, 44 (72.1%) subsequently attained DMR; the median interval from MMR to DMR was 5 months (95% CI, 3–9). Among the 51 pediatric CML-CP patients with evaluable *BCR::ABL1* transcript levels at diagnosis and 3 months, a log-reduction threshold of 0.45 was applied to stratify early molecular responses. Patients with a log-reduction ≥ 0.45 achieved a significantly shorter median time to MMR (11 months; 95% CI: 9–12), compared to those with <0.45 reduction (29 months; 95% CI: 15–NA). Similarly, the median time to DMR was 18 months (95% CI: 12–34) in the ≥0.45 group versus 50 months (95% CI: 49–NA) in the <0.45 group. Patients with a log-reduction ≥ 0.45 exhibited significantly superior outcomes, with 1.5-year MMR rates of 75.5% versus 33.3% (HR, 2.68; 95% CI, 1.18–6.11; *p* = 0.011) and 3-year DMR rates of 76.1% versus 16.7% (HR, 4.02; 95% CI, 1.20–13.45; *p* = 0.014; Figure 3c,d). In multivariable Cox models adjusting for age, baseline BMF, spleen size, TKI switching and ELTS risk, the 3-month *BCR::ABL1* log-reduction < 0.45 (vs. ≥0.45) was not significantly associated with time to MMR (HR, 0.54; 95% CI, 0.17–1.67; *p* = 0.283), but was significantly associated with time to DMR (HR, 0.07; 95% CI, 0.01–0.89; *p* = 0.040). Regardless of the grade of fibrosis, the presence of baseline bone marrow fibrosis (BMF) was also independently associated with time to DMR (HR, 5.55; 95% CI, 1.28–24.09; *p* = 0.022).

### 3.4. Bone Marrow Fibrosis and Genomic Correlates

**Figure 4:**
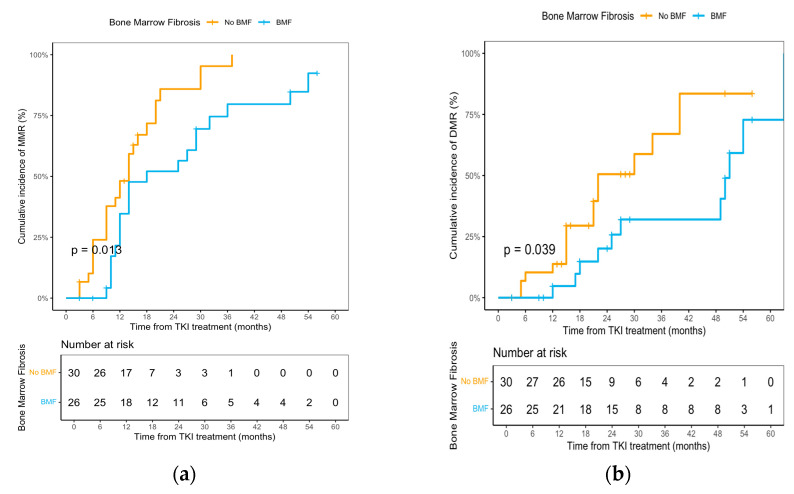
**Cumulative Incidence of MMR and DMR by Bone Marrow Fibrosis in Pediatric CML-CP:** (**a**) MMR; (**b**) DMR.

Bone marrow histology was available for 61 children; 31 had MF-0 and 30 had BMF at diagnosis, including 22 with MF-1, 7 with MF-2 and 1 with MF-3 (Table S2). At baseline, children with BMF had higher white blood cell counts (median 305.1 × 10^9^/L vs. 89.1 × 10^9^/L) and larger spleens (median 13.2 cm vs. 3.0 cm below the left costal margin), whereas platelet counts were slightly lower (median 493 × 10^9^/L vs. 600 × 10^9^/L) compared with those without fibrosis. Among the 30 patients with BMF, follow-up trephine biopsies were available in only two cases: one child with baseline MF-2 showed regression to MF-1 after 3 months of TKI therapy, and another with baseline MF-1 remained MF-1 at 9 months. The presence of BMF was associated with significantly inferior molecular responses: the 1.5-year cumulative incidence of MMR was lower in patients with BMF than in those without fibrosis (54.1% vs. 70.6%; HR, 0.48; 95% CI, 0.26–0.89; *p* = 0.017), and the 3-year cumulative incidence of DMR was likewise lower in the fibrosis group (32.0% vs. 67.0%; HR, 0.44; 95% CI, 0.20–0.99; *p* = 0.039; Figure 4a,b).

Targeted next-generation sequencing was performed in 42 diagnosed children. Six patients had no detectable variants, 13 carried at least one category 1/2 variant, and 25 had only category 3 variants (Figure S4). Variant allele frequencies (VAFs) for individual variants are reported in Supplementary Table S3. *ASXL1* mutations were identified in three patients; two of these (cases #62 and #64) showed poor early molecular responses, failing to achieve 12-month MMR and meeting ELN failure criteria at 3, 6 and 12 months. Case #62 (ELTS high-risk) switched from first-line imatinib to dasatinib after 3 months and remains on TKI therapy, whereas case #64 sequentially received imatinib, dasatinib and olverembatinib, proceeded to allogeneic HSCT at 1 year and is currently in post-transplant remission.

## 4. Discussion

Our findings demonstrate that a rapid early molecular response predicts long-term deep molecular remission in pediatric CML-CP. Children who achieved *BCR::ABL1* ≤ 10% at 3 months had significantly higher cumulative incidences and shorter times to MMR and DMR than those with slower early responses. When early response was quantified kinetically, a 3-month log-reduction ≥ 0.45 from baseline further identified patients with the most favorable outcome, including markedly shorter median time to MMR (11 vs. 29 months) and DMR (18 vs. 50 months).

Our findings mirror the excellent survival reported in other pediatric CML cohorts treated with first-line imatinib. Notably, 5-year OS in our series was 100%, consistent with the >90% OS rates generally observed in children on imatinib. Our 5-year PFS of 95.9% is likewise in line with published PFS estimates (90–95%) in pediatric CML [32,33]. In contrast, EFS and FFS rates are lower, we observed 5-year EFS of 63.1% and FFS of 71.0%, which fall within the broad range reported across studies. For instance, an Indian cohort on imatinib documented 64% EFS at 5 years, whereas a multicenter European study reported 81% EFS [33,34]. This variability likely stems from differences in the definition of events (e.g., loss of response, switching to a second-generation TKI, or transplant) and patient factors such as adherence. Importantly, despite these event rates, long-term survival remains favorable for most patients.

In adult CML data, the 3-month *BCR::ABL1* ≤ 10% threshold as a critical prognostic landmark associated with superior survival and response outcomes [11,35]. In pediatric CML, the importance of the 3-month milestone has likewise been observed: a French multicenter study first showed that children with *BCR::ABL1* ≤ 10% at 3 months had higher 12-month CCyR/MMR rates and improved PFS compared to those >10% [12]. More recently, a Taiwan cohort confirmed that pediatric patients with *BCR::ABL1* ≤ 10% at 3 months were significantly more likely to attain MMR by 1 year [36]. Beyond the conventional 10% landmark, our data emphasize the tempo of early transcript decline as an added discriminator. A log-reduction threshold of 0.45 at 3 months stratified outcomes in our cohort, this accords with adult evidence that greater early log reduction and shorter *BCR::ABL1* halving time predict superior responses. In a large imatinib-treated adult series, >0.61-log reduction at 3 months and halving time ≤ 22 days identified patients with the most favorable course across MMR, MR4.5 and survival endpoints [37]. In our cohort, the median times to MMR was 12 months (95% CI, 10–15). Thus, most children attained MMR within 12–18 months, which is broadly compatible with the international pediatric CML expert panel recommendations that consider failure to achieve MMR by 18–24 months as indicative of primary resistance warranting treatment reassessment or modification [8]. Notably, children with unfavorable early kinetics were more likely to drift beyond this window: among those with *BCR::ABL1* > 10% at 3 months, the median time to MMR was 24 months (95% CI, 14–NA). These findings support the use of early *BCR::ABL1* kinetics to guide treatment reassessment; in particular, children with poor early molecular responses should be considered for finding the cause and may switch to a second-generation TKI, in accordance with current pediatric CML recommendations.

Achieving and maintaining deep molecular response is a key therapeutic objective, particularly in children for whom minimizing lifetime TKI exposure is desirable. In our cohort, 72% of patients who achieved MMR subsequently reached DMR, with a median interval of 5 months from MMR to DMR. Our 5-year cumulative incidences of MMR and DMR are comparable to those in recent pediatric reports [36,38]. That said, recent real-world analyses indicate that although initial molecular response predicts DMR, it does not reliably predict TFR maintenance, implying additional biological determinants of durable off-therapy control [39]. The relationship between early kinetics and TFR deserves specific comment. In adults, *BCR::ABL1* halving time within the first weeks of therapy outperforms static 3-month values in predicting sustained TFR: halving time < 9.35 days was associated with 80% TFR success versus 4% when >21.85 days. Notably, the absolute 3-month *BCR::ABL1* level was not an independent predictor of TFR durability [14].

ELTS was assessable at diagnosis in 73 children, with 55 (75.3%) classified as low risk, 15 (20.5%) as intermediate risk, and 3 (4.1%) as high risk. Patients in the intermediate/high-risk group showed a trend toward poorer long-term outcomes compared with those in the low-risk group (5-year FFS, 53.6% vs. 78.1%; *p* = 0.095; 5-year EFS, 53.6% vs. 71.3%; *p* = 0.23). Data from the international Registry for Chronic Myeloid Leukemia in children and adolescents (I-CML-Ped) study demonstrated significantly higher progression rates among children with elevated ELTS risk scores, with a 5-year PFS of 96% in the low-risk group versus 67% in the high-risk group (*p* < 0.0001) [32]. Notably, OS remained uniformly high across ELTS strata in that study (5-year survival, 97% for the cohort), underscoring that ELTS primarily discriminates progression risk, whereas survival under TKI therapy remains excellent for most pediatric patients. The 5-year EFS rate in our cohort was lower than that reported in some other pediatric series [40]. This discrepancy is likely attributable to methodological differences, as we applied a stricter definition of EFS. Comprehensive event definitions tend to yield lower EFS estimates compared with studies that consider only progression-related events. Differences in risk grouping may also contribute. We stratified patients into two categories—low risk versus combined intermediate/high risk—thereby concentrating all non–low-risk events in a single group, which may inherently result in a lower EFS for that combined category. Despite these differences, our findings support the utility of ELTS as a valuable baseline prognostic tool that complements early molecular response kinetics in pediatric CML.

In our cohort, baseline BMF was associated with slower molecular clearance, reflected by a lower cumulative incidence of MMR at 18 months and DMR at 3 years compared with children without BMF. This pattern is broadly consistent with reports from the imatinib era in adults with CML showing that baseline BMF is associated with higher risks of treatment failure, disease progression and death, and that its adverse prognostic impact may persist even after histologic regression [41]. A recent multicenter study of 925 imatinib-treated adults also found that baseline BMF was associated with worse OS and MR4.5 rates, with separation emerging after 18 months, suggesting that the effect of BMF becomes most apparent at deeper molecular milestones [42]. In contrast, a 2024 cohort reported no significant association between the presence of BMF and initial cytogenetic or molecular response rates, despite worse survival according to BMF grade [43]. The 2025 International Pediatric CML Expert Panel considered bone marrow trephine biopsy at diagnosis to be optional in children because of the low diagnostic yield of myelofibrosis or myelodysplasia and the rarity of blastic transformation detected solely on trephine biopsy [8]. Our data suggest that baseline assessment of marrow fibrosis may provide additional prognostic information; therefore, we recommend performing bone marrow biopsy at diagnosis in pediatric CML. Prospective multicenter pediatric studies are needed to validate these observations.

Targeted sequencing in a subset of our cohort identified additional somatic variants, including *ASXL1* in two children who exhibited poor early responses. In a national registry study of 90 children/adolescents with CML-CP, pathogenic somatic variants were detected in 16%, with *ASXL1* the most frequent; carriers showed trends toward delayed cytogenetic/molecular responses [44]. A pediatric study found 8% of children harbor *ASXL1* mutations at diagnosis, similar to adults, but did not observe a significant impact on response rates or survival [36]. In adults, *ASXL1* mutations at diagnosis predict inferior molecular response under nilotinib, with significantly lower MMR probabilities at 12–24 months [45]. However, the mutational analyses in our study have important limitations. Systematic longitudinal sequencing was not undertaken, precluding a formal assessment of clonal evolution. Moreover, because remission samples and sorted *BCR::ABL1*-negative cells were not routinely sequenced, we cannot reliably distinguish mutations confined to the leukemic clone from those arising in background clonal hematopoiesis.

This study has several limitations. First, it was a retrospective, single-center study with a modest sample size, which may limit the external generalizability of the findings. Second, the cohort was accrued over more than two decades, during which diagnostic work-up, PCR platforms, monitoring schedules, ELN recommendations and the overall therapeutic landscape of CML evolved. These era-related changes may have introduced heterogeneity in response assessment and management and should be taken into account when interpreting the results. Third, there was heterogeneity in TKI treatment over time. Although initial therapy was highly uniform, with imatinib used as first-line treatment in almost all children such that 3-month early molecular response assessments were largely obtained under imatinib-based therapy, 25 patients subsequently switched to second- or third-generation TKIs, usually for suboptimal response or intolerance. Such treatment changes may have influenced the probability and timing of achieving MMR and DMR and may have introduced treatment selection bias. The study was not adequately powered to support formal comparative analyses between individual TKIs or to fully disentangle treatment era and treatment selection effects from other prognostic factors. Fourth, analyses of 3-month early molecular response were limited to the 57 patients with an evaluable 3-month molecular assessment. The absence of 3-month data in the remaining patients may introduce selection bias and weakens the strength of inferences regarding early molecular predictors of subsequent MMR and DMR, so these findings should be interpreted with appropriate caution.

## 5. Conclusions

In summary, this study highlights that a rapid early decline in *BCR::ABL1* transcript levels is a strong predictor of deep molecular remission in pediatric CML-CP. Children achieving *BCR::ABL1* ≤ 10% or a log-reduction ≥ 0.45 at 3 months are significantly more likely to attain subsequent MMR and DMR. These findings suggest that early *BCR::ABL1* kinetics can be used as a practical trigger for treatment reassessment in pediatric CML. In children who have *BCR::ABL1* > 10% or a 3-month log-reduction < 0.45, a suboptimal early molecular response should prompt a systematic review of treatment adherence and TKI exposure. In parallel, baseline risk features such as ELTS risk category and bone marrow fibrosis should be revisited to identify children with a higher underlying risk profile. For patients with persistently slow early molecular decline despite optimization of these factors, and especially those with additional high-risk characteristics, an early switch to a second-generation TKI could be considered.

## Figures and Tables

**Table 1:** Patient characteristics of the overall cohort.

Characteristic	*N* (%)
**Age, years, median (range)**	9.2 (1.0–17.0)
**Sex**	
Male	64 (72.7)
Female	24 (27.3)
Leukocyte count × 10^9^/L, median (range)	156.8 (23.9–709.6)
Platelet count × 10^9^/L, median (range)	529 (187–3369)
Hemoglobin, g/L, median (range)	98 (61–151)
Blood basophils, %, median (range)	4 (0–12)
**Symptoms at diagnosis**	
Fatigue	24 (27.3)
Fever	28 (31.8)
Bleeding tendencies	9 (10.2)
Abdominal distension/discomfort	17 (19.3)
Weight loss	8 (9.1)
Asymptomatic	25 (28.4)
Bone pain	8 (9.1)
Priapism	2(2.3)
**Clinical signs at diagnosis**	
Hepatomegaly	25 (28.4)
Splenomegaly	61 (69.3)
Spleen size below costal margin, cm, median (range)	9.0 (1.0–25.3)
**ELTS**	
Low-risk	55 (62.5)
Intermediate-risk	15 (17.0)
High-risk	3 (3.4)
Unknown	15 (7.0)
**Ph^+^ ACAs**	
Yes	5 (5.7)
No	75 (85.2)
Unknown	8 (9.1)

ELTS, European Treatment and Outcome Study Long-Term Survival score; ACAs, additional chromosomal aberrations.

**Table 2:** Molecular Response in Pediatric CML-CP on TKI Therapy.

Time Since TKI Initiation	Evaluable Patients, *n*	Molecular Response, *n* (%)		
		Optimal	Warning	Failure
**3** **m**	57	39 (68.4)	12 (21.1)	6 (10.5)
**6** **m**	50	30 (60.0)	16 (32.0)	4 (8.0)
**12** **m**	52	28 (53.8)	14 (26.9)	10 (19.2)

TKI, Tyrosine Kinase Inhibitor.

## Data Availability

The datasets presented in this article are not readily available because the data are part of an ongoing multicenter study and are subject to collaborative restrictions. Requests to access the datasets should be directed to the corresponding author, Wenyu Yang (yangwenyu@ihcams.ac.cn).

## Supplementary Materials

The following supporting information can be downloaded at: https://www.mdpi.com/article/10.3390/cancers17243994/s1, Figure S1: TKI Switching Pathways in Pediatric CML-CP; Figure S2: (a) Failure-free survival of pediatric patients with CML-CP stratified by ELTS risk groups (b) Event-free survival of pediatric patients with CML-CP stratified by ELTS risk groups; Figure S3: EFS and FFS stratified by 3-month *BCR::ABL1* transcript dynamics; Figure S4: Mutation spectrum of CML patients; Table S1: Baseline and Treatment Characteristics of Pediatric CML-CP With TKI Switching; Table S2: Baseline characteristics in pediatric CML patients with bone marrow fibrosis; Table S3: Clinical Characteristics and Molecular Response of Pediatric CML-CP Patients with Class I/II Hematologic Malignancy-Related Mutations.

## Abbreviations

The following abbreviations are used in this manuscript:

CML	Chronic myeloid leukemia
TKI	Tyrosine kinase inhibitor
EMR	Early molecular response
MMR	Major molecular response
DMR	Deep molecular remission
TFR	Treatment-free remission
ELN	European Leukemia Net
ELTS	EUTOS long-term survival
BMF	Bone marrow fibrosis
ACA	Additional chromosomal abnormality
qRT-PCR	Quantitative real-time polymerase chain reaction
PCR	Polymerase chain reaction
IS	International Scale
WBC	White blood cell
CHR	Complete hematologic response
CCyR	Complete cytogenetic response
Ph+	Philadelphia chromosome–positive
OS	Overall survival
PFS	Progression-free survival
EFS	Event-free survival
FFS	Failure-free survival
AP	Accelerated phase
BP	Blast phase
CI	Confidence interval
HSCT	Hematopoietic stem-cell transplantation
MR4	Molecular response 4
MR4.5	Molecular response 4.5
MR5	Molecular response 5

## References

[1] Athale U., Hijiya N., Patterson B.C., Bergsagel J., Andolina J.R., Bittencourt H., Schultz K.R., Burke M.J., Redell M.S., Kolb E.A. (2019). Management of chronic myeloid leukemia in children and adolescents: Recommendations from the Children’s Oncology Group CML Working Group. Pediatr. Blood Cancer.

[2] Hijiya N., Suttorp M. (2019). How I treat chronic myeloid leukemia in children and adolescents. Blood.

[3] Hijiya N., Schultz K.R., Metzler M., Millot F., Suttorp M. (2016). Pediatric chronic myeloid leukemia is a unique disease that requires a different approach. Blood.

[4] Ries L.A.G., Smith M.A., Gurney J.G., Linet M., Tamra T., Young J.L., Bunin G.R. (1999). Cancer Incidence and Survival Among Children and Adolescents: United States SEER Program 1975–1995; SEER Program.

[5] Shima H., Tokuyama M., Tanizawa A., Tono C., Hamamoto K., Muramatsu H., Watanabe A., Hotta N., Ito M., Kurosawa H. (2011). Distinct impact of imatinib on growth at prepubertal and pubertal ages of children with chronic myeloid leukemia. J. Pediatr..

[6] Millot F., Guilhot J., Baruchel A., Petit A., Leblanc T., Bertrand Y., Mazingue F., Lutz P., Vérité C., Berthou C. (2014). Growth deceleration in children treated with imatinib for chronic myeloid leukaemia. Eur. J. Cancer.

[7] Hochhaus A., Baccarani M., Silver R.T., Schiffer C., Apperley J.F., Cervantes F., Clark R.E., Cortes J.E., Deininger M.W., Guilhot F. (2020). European LeukemiaNet 2020 recommendations for treating chronic myeloid leukemia. Leukemia.

[8] Millot F., Ampatzidou M., Moulik N.R., Tewari S., Elhaddad A., Hammad M., Pichler H., Lion T., Tragiannidis A., Shima H. (2025). Management of children and adolescents with chronic myeloid leukemia in chronic phase: International pediatric chronic myeloid leukemia expert panel recommendations. Leukemia.

[9] Marin D., Ibrahim A.R., Lucas C., Gerrard G., Wang L., Szydlo R.M., Clark R.E., Apperley J.F., Milojkovic D., Bua M. (2012). Assessment of BCR-ABL1 transcript levels at 3 months is the only requirement for predicting outcome for patients with chronic myeloid leukemia treated with tyrosine kinase inhibitors. J. Clin. Oncol..

[10] Hughes T.P., Saglio G., Kantarjian H.M., Guilhot F., Niederwieser D., Rosti G., Nakaseko C., De Souza C.A., Kalaycio M.E., Meier S. (2014). Early molecular response predicts outcomes in patients with chronic myeloid leukemia in chronic phase treated with frontline nilotinib or imatinib. Blood.

[11] Hanfstein B., Müller M.C., Hehlmann R., Erben P., Lauseker M., Fabarius A., Schnittger S., Haferlach C., Göhring G., Proetel U. (2012). Early molecular and cytogenetic response is predictive for long-term progression-free and overall survival in chronic myeloid leukemia (CML). Leukemia.

[12] Millot F., Guilhot J., Baruchel A., Petit A., Bertrand Y., Mazingue F., Lutz P., Vérité C., Berthou C., Galambrun C. (2014). Impact of early molecular response in children with chronic myeloid leukemia treated in the French Glivec phase 4 study. Blood.

[13] Branford S., Yeung D.T., Parker W.T., Roberts N.D., Purins L., Braley J.A., Altamura H.K., Yeoman A.L., Georgievski J., Jamison B.A. (2014). Prognosis for patients with CML and >10% BCR-ABL1 after 3 months of imatinib depends on the rate of BCR-ABL1 decline. Blood.

[14] Shanmuganathan N., Pagani I.S., Ross D.M., Park S., Yong A.S.M., Braley J.A., Altamura H.K., Hiwase D.K., Yeung D.T., Kim D.W. (2021). Early BCR-ABL1 kinetics are predictive of subsequent achievement of treatment-free remission in chronic myeloid leukemia. Blood.

[15] Iriyama N., Fujisawa S., Yoshida C., Wakita H., Chiba S., Okamoto S., Kawakami K., Takezako N., Kumagai T., Inokuchi K. (2015). Shorter halving time of BCR-ABL1 transcripts is a novel predictor for achievement of molecular responses in newly diagnosed chronic-phase chronic myeloid leukemia treated with dasatinib: Results of the D-first study of Kanto CML study group. Am. J. Hematol..

[16] Fava C., Rege-Cambrin G., Dogliotti I., Gottardi E., Berchialla P., Di Gioacchino B., Crasto F., Lorenzatti R., Volpengo A., Daraio F. (2016). Early BCR-ABL1 reduction is predictive of better event-free survival in patients with newly diagnosed chronic myeloid leukemia treated with any tyrosine kinase inhibitor. Clin. Lymphoma Myeloma Leuk..

[17] Pennisi M.S., Stella S., Vitale S.R., Puma A., Di Gregorio S., Romano C., Tirrò E., Massimino M., Antolino A., Siragusa S. (2019). BCR-ABL1 doubling-times and halving-times may predict CML response to tyrosine kinase inhibitors. Front. Oncol..

[18] Cai Z., Jia X., Zi J., Song H., Wang S., McGrath M., Zhao L., Song C., Ge Z. (2020). BCR-ABL1 transcript decline ratio combined BCR-ABL1IS as a precise predictor for imatinib response and outcome in the patients with chronic myeloid leukemia. J. Cancer.

[19] Shima H., Kada A., Tanizawa A., Sato I., Tono C., Ito M., Yuza Y., Watanabe A., Kamibeppu K., Uryu H. (2022). Discontinuation of tyrosine kinase inhibitors in pediatric chronic myeloid leukemia. Pediatr. Blood Cancer.

[20] Millot F., Suttorp M., Ragot S., Leverger G., Dalle J.H., Thomas C., Cheikh N., Nelken B., Poirée M., Plat G. (2021). Discontinuation of imatinib in children with chronic myeloid leukemia: A study from the international registry of childhood CML. Cancers.

[21] Dave D., Trivedi M., Doctor C., Parikh B., Panchal H.P., Yadav R. (2025). Impact of Molecular and Cytogenetic Responses on Long-Term Outcomes in Children and Adolescents with Chronic Myeloid Leukemia: A Retrospective Study from India. Cureus.

[22] Stella S., Zammit V., Vitale S.R., Pennisi M.S., Massimino M., Tirrò E., Forte S., Spitaleri A., Antolino A., Siracusa S. (2019). Clinical Implications of Discordant Early Molecular Responses in CML Patients Treated with Imatinib. Int. J. Mol. Sci..

[23] Narlı Özdemir Z., Kılıçaslan N.A., Yılmaz M., Eşkazan A.E. (2023). Guidelines for the treatment of chronic myeloid leukemia from the NCCN and ELN: Differences and similarities. Int. J. Hematol..

[24] Baccarani M., Cortes J., Pane F., Niederwieser D., Saglio G., Apperley J., Cervantes F., Deininger M., Gratwohl A., Guilhot F. (2009). Chronic myeloid leukemia: An update of concepts and management recommendations of European LeukemiaNet. J. Clin. Oncol..

[25] Baccarani M., Deininger M.W., Rosti G., Hochhaus A., Soverini S., Apperley J.F., Cervantes F., Clark R.E., Cortes J.E., Guilhot F. (2013). European LeukemiaNet recommendations for the management of chronic myeloid leukemia: 2013. Blood.

[26] Baccarani M., Saglio G., Goldman J., Hochhaus A., Simonsson B., Appelbaum F., Apperley J., Cervantes F., Cortes J., Deininger M. (2006). Evolving concepts in the management of chronic myeloid leukemia: Recommendations from an expert panel on behalf of the European LeukemiaNet. Blood.

[27] van Dongen J.J., Macintyre E.A., Gabert J.A., Delabesse E., Rossi V., Saglio G., Gottardi E., Rambaldi A., Dotti G., Griesinger F. (1999). Standardized RT-PCR analysis of fusion gene transcripts from chromosome aberrations in acute leukemia for detection of minimal residual disease. Report of the BIOMED-1 Concerted Action: Investigation of minimal residual disease in acute leukemia. Leukemia.

[28] Beillard E., Pallisgaard N., van der Velden V.H., Bi W., Dee R., van der Schoot E., Delabesse E., Macintyre E., Gottardi E., Saglio G. (2003). Evaluation of candidate control genes for diagnosis and residual disease detection in leukemic patients using ‘real-time’ quantitative reverse-transcriptase polymerase chain reaction (RQ-PCR)—A Europe against cancer program. Leukemia.

[29] White H.E., Salmon M., Albano F., Andersen C.S.A., Balabanov S., Balatzenko G., Barbany G., Cayuela J.M., Cerveira N., Cochaux P. (2022). Standardization of molecular monitoring of CML: Results and recommendations from the European treatment and outcome study. Leukemia.

[30] Pfirrmann M., Baccarani M., Saussele S., Guilhot J., Cervantes F., Ossenkoppele G., Hoffmann V.S., Castagnetti F., Hasford J., Hehlmann R. (2016). Prognosis of long-term survival considering disease-specific death in patients with chronic myeloid leukemia. Leukemia.

[31] Ghosh K., Shome D.K., Kulkarni B., Ghosh M.K., Ghosh K. (2023). Fibrosis and bone marrow: Understanding causation and pathobiology. J. Transl. Med..

[32] Millot F., Guilhot J., Suttorp M., Güneş A.M., Sedlacek P., De Bont E., Li C.K., Kalwak K., Lausen B., Culic S. (2017). Prognostic discrimination based on the EUTOS long-term survival score within the International Registry for Chronic Myeloid Leukemia in children and adolescents. Haematologica.

[33] Ganguly S., Pushpam D., Mian A., Chopra A., Gupta R., Bakhshi S. (2020). Real-world Experience of Imatinib in Pediatric Chronic Phase Chronic Myeloid Leukemia: A Single-center Experience from India. Clin. Lymphoma Myeloma Leuk..

[34] Janeczko-Czarnecka M., Krawczuk-Rybak M., Karpińska-Derda I., Niedźwiecki M., Musioł K., Ćwiklińska M., Drabko K., Mycko K., Ociepa T., Pawelec K. (2018). Imatinib in the treatment of chronic myeloid leukemia in children and adolescents is effective and well tolerated: Report of the Polish Pediatric Study Group for the Treatment of Leukemias and Lymphomas. Adv. Clin. Exp. Med..

[35] Branford S., Kim D.W., Soverini S., Haque A., Shou Y., Woodman R.C., Kantarjian H.M., Martinelli G., Radich J.P., Saglio G. (2012). Initial molecular response at 3 months may predict both response and event-free survival at 24 months in imatinib-resistant or -intolerant patients with Philadelphia chromosome-positive chronic myeloid leukemia in chronic phase treated with nilotinib. J. Clin. Oncol..

[36] Liu H.C., Kuo M.C., Wu K.H., Chen T.Y., Chen J.S., Wang M.C., Lin T.L., Yang Y., Ma M.C., Wang P.N. (2023). Children with chronic myeloid leukaemia treated with front-line imatinib have a slower molecular response and comparable survival compared with adults: A multicenter experience in Taiwan. Br. J. Cancer.

[37] Zhang J., Wang Y., Wang J., Hu J., Chen S., Jin J., Liu T., Zhou J., Hu Y., Ma D. (2018). Early BCR-ABL1 decline in imatinib-treated patients with chronic myeloid leukemia: Results from a multicenter study of the Chinese CML alliance. Blood Cancer J..

[38] Millot F., Baruchel A., Guilhot J., Petit A., Leblanc T., Bertrand Y., Mazingue F., Lutz P., Vérité C., Berthou C. (2011). Imatinib is effective in children with previously untreated chronic myelogenous leukemia in early chronic phase: Results of the French national phase IV trial. J. Clin. Oncol..

[39] Saugues S., Lambert C., Daguenet E., Roth-Guepin G., Huguet F., Cony-Makhoul P., Ansah H.J., Escoffre-Barbe M., Turhan A., Rousselot P. (2024). The initial molecular response predicts the deep molecular response but not treatment-free remission maintenance in a real-world chronic myeloid leukemia cohort. Haematologica.

[40] Zhang X.S., Gale R.P., Huang X.J., Jiang Q. (2022). Is the Sokal or EUTOS long-term survival (ELTS) score a better predictor of responses and outcomes in persons with chronic myeloid leukemia receiving tyrosine-kinase inhibitors?. Leukemia.

[41] Buesche G., Ganser A., Schlegelberger B., von Neuhoff N., Gadzicki D., Hecker H., Bock O., Frye B., Kreipe H. (2007). Marrow fibrosis and its relevance during imatinib treatment of chronic myeloid leukemia. Leukemia.

[42] Zeng T., Yang X., Wang Y., Wu D., Feng W., Lu Y., Zhu X., Liu L., Zhou M., Zhang L. (2024). Myelofibrosis predicts deep molecular response 4.5 in chronic myeloid leukaemia patients initially treated with imatinib: An extensive, multicenter and retrospective study to develop a prognostic model. Clin. Transl. Med..

[43] Pepeler M.S., Tıglıoglu M., Dagdas S., Ozhamamcıoglu E., Han U., Albayrak A., Aydın M.S., Korkmaz G., Pamukcuoğlu M., Ceran F. (2024). Prognostic Impact of Bone Marrow Fibrosis and Effects of Tyrosine Kinase Inhibitors on Bone Marrow Fibrosis in Chronic Myeloid Leukemia. Clin. Lymphoma Myeloma Leuk..

[44] Behrens Y.L., Gaschler L., Nienhold R., Reinkens T., Schirmer E., Knöß S., Strasser R., Sembill S., Wotschofsky Z., Suttorp M. (2024). Somatic variant profiling in chronic phase pediatric chronic myeloid leukemia. Haematologica.

[45] Schönfeld L., Rinke J., Hinze A., Nagel S.N., Schäfer V., Schenk T., Fabisch C., Brümmendorf T.H., Burchert A., le Coutre P. (2022). ASXL1 mutations predict inferior molecular response to nilotinib treatment in chronic myeloid leukemia. Leukemia.

