# Supplementary material for: Early BCR::ABL1 Reduction as a Predictor of Deep Molecular Response in Pediatric Chronic-Phase Chronic Myeloid Leukemia
Source: Cancers (Basel). 2025 Dec 15;17(24):3994. doi: 10.3390/cancers17243994 (PMC12731570; doi:10.3390/cancers17243994)
Supplement: Supplementary file 1 [file cancers-17-03994-s001.zip › cancers-4010002-supplementary.docx]

Supplementary Materials: Early *BCR::ABL1* Reduction as A Predictor of Deep Molecular Response in Pediatric
Chronic-Phase Chronic Myeloid Leukemia

Xingchen Wang, Wenbin An , Chenmeng Liu, Bang Zhang, Yunlong Chen, Yang Wan, Xiaolan Li, Lipeng Liu, Fang Liu, Li Zhang, Yao Zou, Xiaojuan Chen, Yumei Chen, Ye Guo, Tianyuan Hu, Yingchi Zhang, Xiaofan Zhu and Wenyu Yang


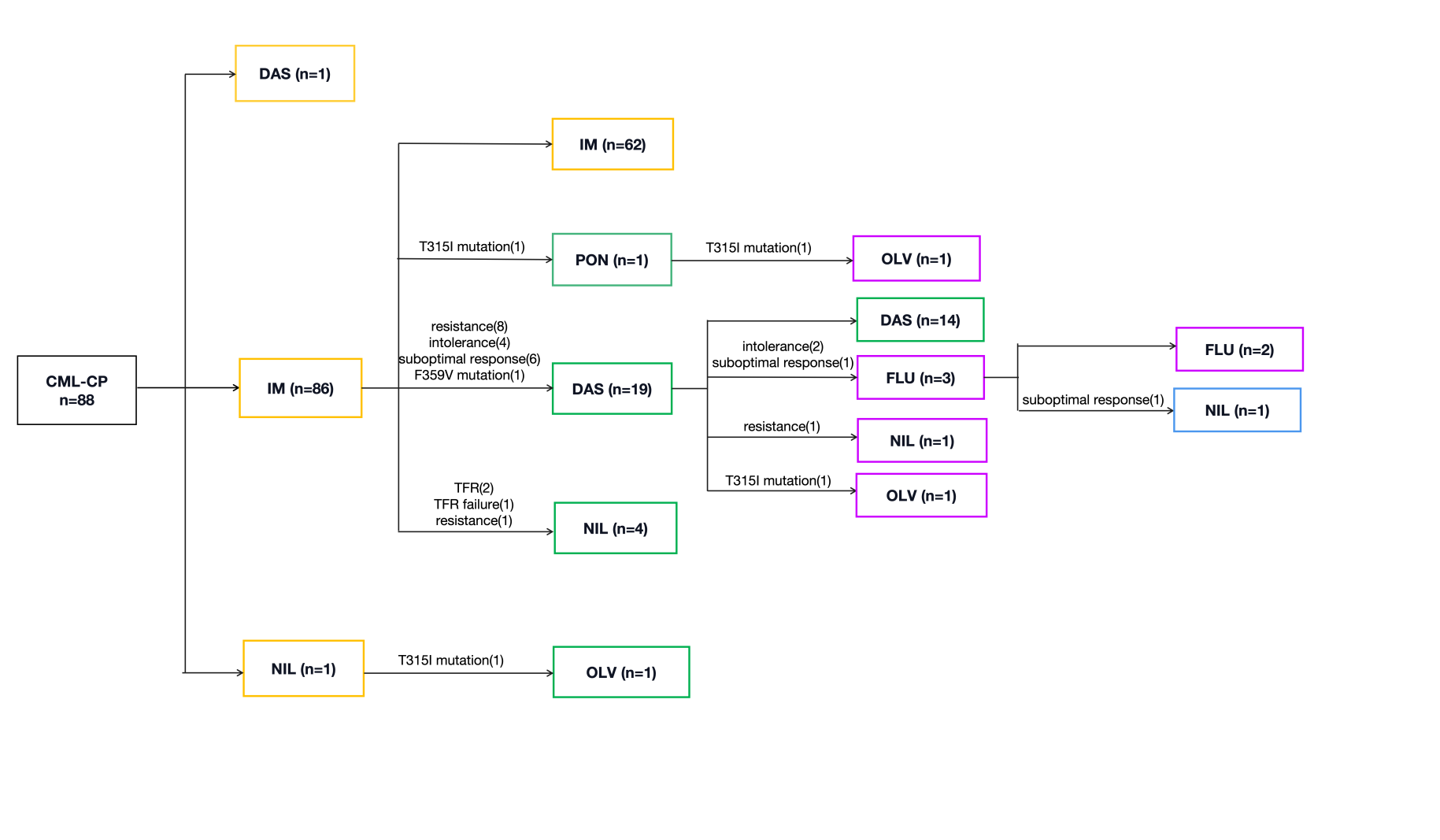


**Figure S1.** TKI Switching Pathways in Pediatric CML-CP**.** IM, Imatinib; DAS, Dasatinib; NIL, Nilotinib; FLU, Flumatinib; PON, Ponatinib; OLV, Olverembatinib.

| **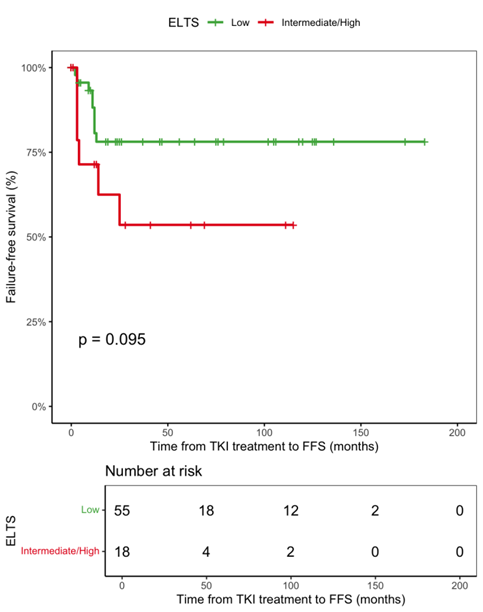** | **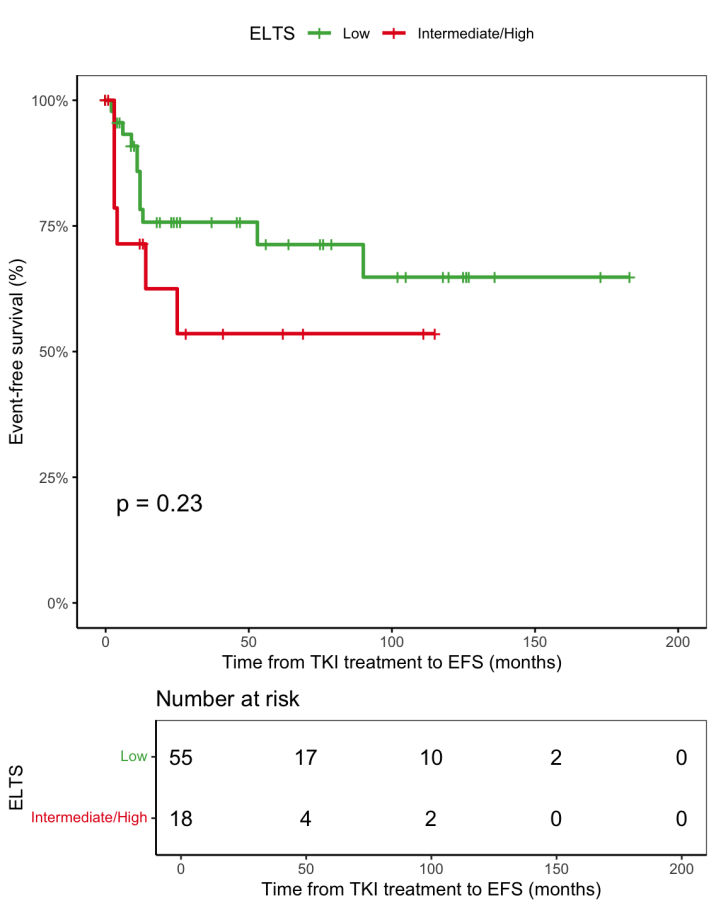** |
| --- | --- |
| (**a**) | (**b**) |

**Figure S2.** (**a**) Failure-free survival of pediatric patients with CML-CP stratified by ELTS risk groups (**b**) Event-free survival of pediatric patients with CML-CP stratified by ELTS risk groups.

| **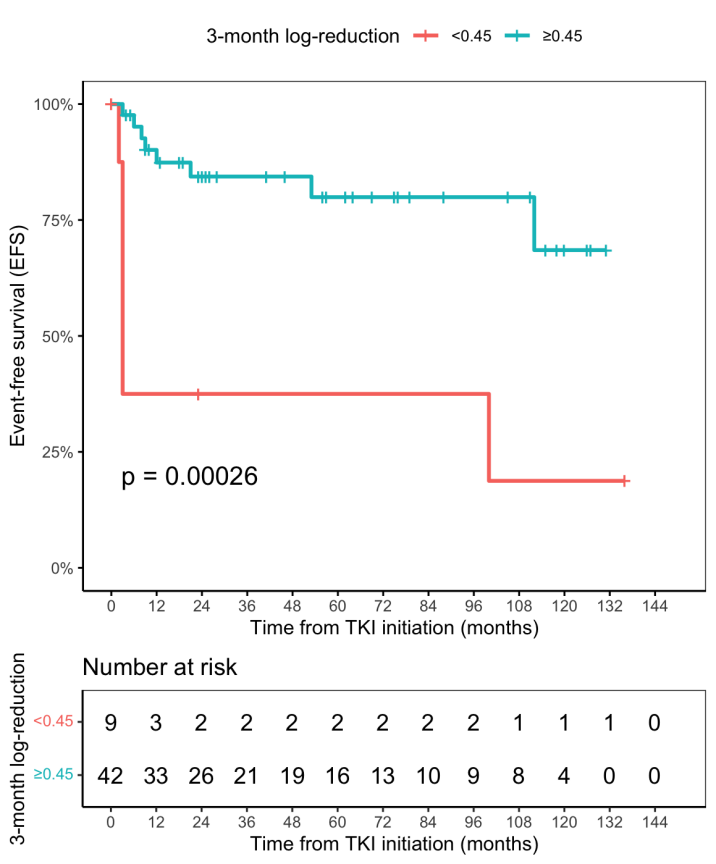** | **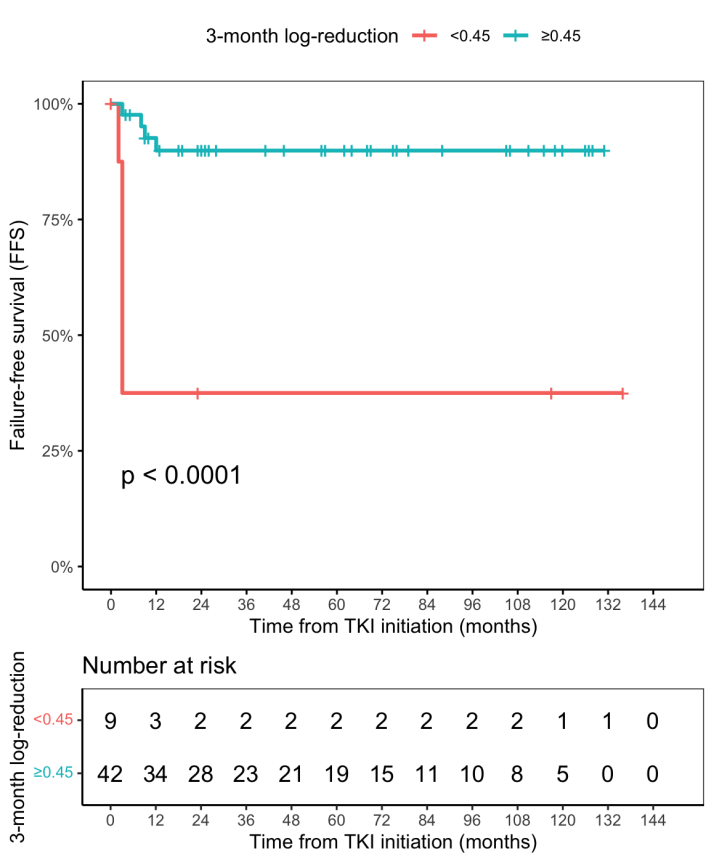** |
| --- | --- |
| (**a**) | (**b**) |

**Figure S3.** EFS and FFS stratified by 3-month *BCR::ABL1* transcript dynamics**.**


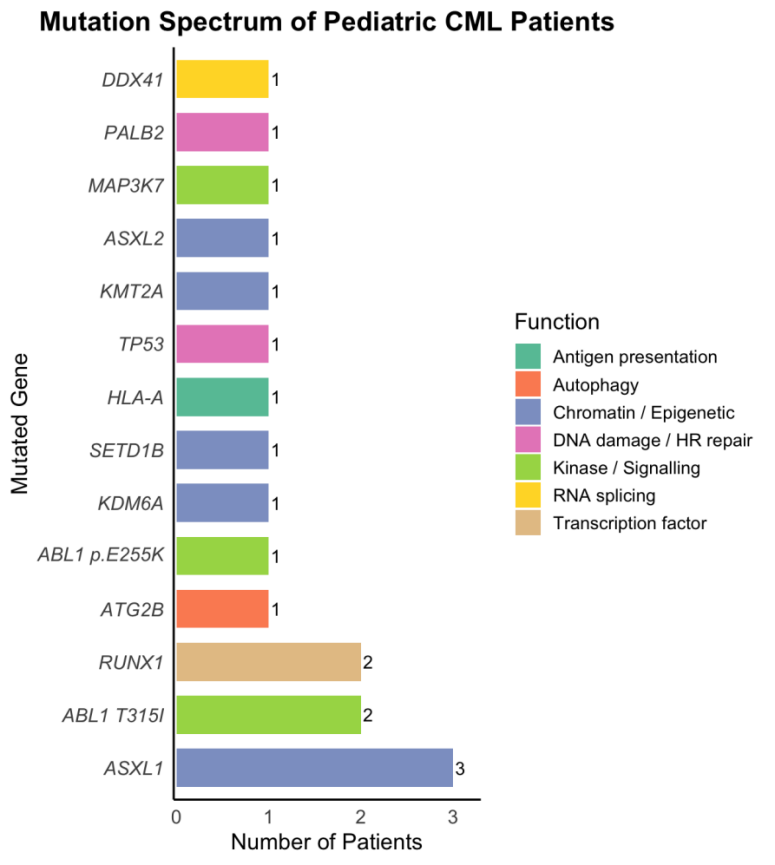


**Figure S4.** Mutation spectrum of CML patients**.**

After passing quality control, all identified variants were described according to HGVS nomenclature and annotated using multiple databases (COSMIC, ClinVar, HGMD, ExAC, PolyPhen-2, dbSNP, etc.) to facilitate further screening. The remaining variants were then manually reviewed and categorized into three classes based on the AMP/ASCO/CAP guidelines for somatic variant interpretation.

Category 1 consisted of known “hotspot” mutations strongly associated with the disease. This included variants explicitly documented as disease-defining in consensus guidelines (e.g., WHO, NCCN, ELN, ICC) or in authoritative literature, as well as mutations in key functional domains consistent with known pathogenic mechanisms of the gene. Category 2 encompassed mutations potentially associated with the disease. For example, this category captured likely splice-site mutations predicted to disrupt mRNA splicing, and rare variants – absent or with frequency <0.01 in population databases – that were predicted to be deleterious by at least two in silico predictive tools, provided their variant allele frequency (VAF) lay outside the near-heterozygous (45–55%) or near-homozygous/clonal (95–100%) ranges. Category 3 included all other detected variants that did not meet criteria for Category 1 or 2. These were generally variants of uncertain significance, such as rare variants predicted to be tolerated by the majority of computational prediction algorithms (≥4 tools) with VAF outside the 45–55% and 95–100% ranges, as well as rare variants with VAF in those (~50% or ~100%) ranges that had no benign classification in cancer databases but were flagged as potentially deleterious by at least one prediction tool. This classification scheme ensured that well-established pathogenic mutations (Category 1) and likely pathogenic variants (Category 2) were distinguished from other variants of unclear significance (Category 3) in our analysis.

**Table S1.** Baseline and Treatment Characteristics of Pediatric CML-CP With TKI Switching**.**

| **UPN** | **Age(y)/sex** | **ELTS** | **First-Line** | **Time** | **Second-Line** | **Time** | **Third-Line** | **Time** | **Fourth-Line** | **Time** | **Reason for Switch** | **At Last Follow Up** |
| --- | --- | --- | --- | --- | --- | --- | --- | --- | --- | --- | --- | --- |
| 16 | 13.0/M | NA | Imatinib | 84m | Nilotinib | 12m | **-** |  | **-** |  | TFR attempt* | Imatinib, MR5 |
| 24 | 11.4/F | L | Imatinib | 102m | Dasatinib | 7m | **-** |  | **-** |  | suboptimal response | Dasatinib, MR5 |
| 25 | 9.4/M | NA | Imatinib | 102m | Nilotinib | 13m | - |  | - |  | TFR failure | Nilotinib, MR5 |
| 30 | 3.6/M | L | Imatinib | 12m | Nilotinib | 99m | - |  | - |  | resistance | Nilotinib, MR2 |
| 31 | 7.3/M | NA | Imatinib | 30m | Nilotinib | 59m | - |  | - |  | TFR attempt* | in TFR , MR5 |
| 38 | 7.7/M | L | Imatinib | 12m | Dasatinib | 66m | - |  | - |  | suboptimal response | Dasatinib, MR5 |
| 39 | 1.0/M | NA | Imatinib | 49m | Dasatinib | 1m | - |  | - |  | resistance | Imatinib, MMR |
| 44 | 1.7/F | L | Imatinib | 12m | Dasatinib | 24m | Nilotinib | 36m | - |  | resistance | Nilotinib, MMR |
| 45 | 8.6/F | L | Imatinib | 43m | Dasatinib | 25m | - |  | - |  | resistance | Dasatinib, MMR |
| 46 | 1.9/M | L | Imatinib | 32m | Dasatinib | 36m | - |  | - |  | resistance | Dasatinib, MR2 |
| 49 | 8.4/M | I | Imatinib | 8m | Dasatinib | 8m | Flumatinib | 8m | Nilotinib | 34m | suboptimal response | Nilotinib, MR2 |
| 52 | 6.1/M | NA | Imatinib | 4m | Ponatinib | 51m | - |  | - |  | T315I mutation | Ponatinib, MR5 |
| 53 | 4.9/M | L | Imatinib | 12m | Dasatinib | 18m | Flumatinib | 6m | - |  | intolerance | Off TKI, unknown |
| 60 | 10.7/M | NA | Imatinib | 11m | Dasatinib | 25m | - |  | - |  | resistance | Dasatinib, MR5 |
| 61 | 11.8/M | L | Imatinib | 26m | Dasatinib | 14m | - |  | - |  | resistance | Dasatinib, MMR |
| 62 | 12.4/F | H | Imatinib | 3m | Dasatinib | 29m | - |  | - |  | resistance | Dasatinib, MMR |
| 63 | 10.0/M | I | Imatinib | 12m | Dasatinib | 17m | - |  | - |  | suboptimal response | Dasatinib, MMR |
| 64 | 6.1/F | NA | Imatinib | 5m | Dasatinib | 3m | Olverembatinib | 20m | - |  | resistance/ T315I mutation | Olverembatinib, MR5 |
| 68 | 8.5/M | L | Imatinib | 7m | Dasatinib | 19m | - |  | - |  | intolerance | Dasatinib, MMR |
| 69 | 13.4/M | L | Imatinib | 4m | Dasatinib | 20m | - |  | - |  | intolerance | Dasatinib, MMR |
| 70 | 9.9/M | L | Imatinib | 21m | Dasatinib | 4m | - |  | - |  | suboptimal response | Dasatinib, MMR |
| 71 | 13.6/M | L | Imatinib | 6m | Dasatinib | 4m | Flumatinib | 15m | - |  | intolerance | Flumatinib, MR5 |
| 74 | 8.8/M | L | Imatinib | 12m | Dasatinib | 8m | - |  | - |  | suboptimal response | Dasatinib, MMR |
| 75 | 9.8/M | L | Imatinib | 14m | Dasatinib | 5m | - |  | - |  | F359V mutation | Dasatinib, MR5 |
| 80 | 12.1/M | NA | Nilotinib | 8m | Olverembatinib | 9m | - |  | - |  | T315I mutation | Olverembatinib, MR5 |

* Patients underwent an elective switch from imatinib to a second-generation TKI to achieve deep molecular response and enable a future TFR attempt.

**Table S2.** Baseline characteristics in pediatric CML patients with bone marrow fibrosis**.**

| **UPN** | **BMF garde** | **Age(y)/sex** |  | **ELTS** | **Spleen size (cm)*** | **WBC (×10^9^/L)** | **Hb (g/L)** | **PLT (×10^9^/L)** | **Last BMF Grade** | **Last Follow-Up Status** |
| --- | --- | --- | --- | --- | --- | --- | --- | --- | --- | --- |
| 1 | 1 | 5.0/F |  | L | 6 | 135.2 | 89 | 790 | NA | MR5 |
| 2 | 3 | 9.1/M |  | L | 10 | 428.2 | 65 | 2174 | NA | MMR |
| 3 | 2 | 15.5/M |  | L | 17.5 | 305.48 | 79 | 876 | NA | Lost to follow up |
| 5 | 1 | 11.7/F |  | H | 20 | 135.69 | 94 | 777 | NA | MR5 |
| 6 | 1 | 13.3/M |  | L | 12 | 311.8 | 85 | 1592 | NA | MR5 |
| 7 | 1 | 2.9/M |  | NA | 0 | 35.35 | 109 | 388 | NA | MR5 |
| 8 | 2 | 10.3/M |  | I | 13.1 | 444.14 | 74 | 197 | NA | Post-transplant remission |
| 9 | 1 | 8.2/M |  | I | 20 | 265.71 | 105 | 428 | NA | MR5 |
| 10 | 1 | 4.6/F |  | L | 0 | 156.77 | 94 | 239 | NA | MMR |
| 11 | 2 | 6.9/F |  | L | 15 | 709.6 | 72 | 508 | NA | MR5 |
| 25 | 1 | 9.4/M |  | L | 11 | 409.54 | 70 | 529 | NA | MR5 |
| 29 | 1 | 13.9/F |  | I | 18.5 | 457.61 | 80 | 514 | NA | MR5 |
| 31 | 2 | 7.3/M |  | NA | 8 | 32.72 | 83 | 883 | NA | MR5 |
| 33 | 1 | 11.1/M |  | H | 16.9 | 468.54 | 70 | 288 | NA | MR5 |
| 34 | 1 | 6.6/F |  | L | 0 | 48.23 | 101 | 1487 | NA | MR5 |
| 35 | 1 | 12.3/M |  | I | 15.4 | 529.42 | 99 | 276 | NA | MR5 |
| 42 | 1 | 4.8/M |  | L | 1.4 | 27.36 | 107 | 3369 | NA | MR5 |
| 43 | 1 | 12.5/F |  | I | 17.8 | 511.49 | 76 | 277 | NA | MR5 |
| 48 | 1 | 10.0/F |  | I | 13 | 432.53 | 76 | 506 | NA | MR5 |
| 49 | 2 | 8.4/M |  | I | 17.4 | 382.16 | 72 | 419 | NA | MR2 |
| 51 | 1 | 12.6/M |  | I | 13.5 | 304.8 | 78 | 322 | NA | MMR |
| 52 | 1 | 6.1/M |  | NA | NA | 134.4 | 92 | 236 | NA | Post-transplant remission |
| 53 | 1 | 4.9/M |  | L | 17.8 | 452.9 | 66 | 719 | NA | Lost to follow up |
| 57 | 1 | 5.9/M |  | I | 13.2 | 488.56 | 65 | 228 | NA | MMR |
| 60 | 1 | 10.7/M |  | NA | NA | 227.63 | 112 | 443 | NA | MR5 |
| 62 | 2 | 12.4/F |  | H | 22 | 246.36 | 65 | 206 | 1 (3m) | MMR |
| 64 | 1 | 6.1/F |  | NA | NA | 126.92 | 102 | 453 | NA | Post-transplant remission |
| 66 | 1 | 10.0/F |  | L | 5 | 153.64 | 101 | 625 | 1 (9m) | MR4 |
| 83 | 2 | 12.8/M |  | I | 16 | 439.98 | 61 | 347 | NA | MMR |
| 85 | 1 | 13.4/F |  | L | 9 | 147.7 | 115 | 1701 | NA | MR5 |

BMF, bone marrow fibrosis; ELTS, EUTOS long-term survival; WBC, white blood cell count; Hb, hemoglobin; PLT, platelet count; *Spleen size (below left costal margin).

**Table S3.** Clinical Characteristics and Molecular Response of Pediatric CML-CP Patients with Class I/II Hematologic Malignancy-Related Mutations**.**

| **UPN** | **Age(y)/sex** | **Gene Mutation 1 (VAF, %)** | **Gene Mutation 2 (VAF, %)** | **Gene Mutation 3 (VAF, %)** | **EMR** | **12m MMR** | **MMR/Time(m)** | **MR4/Time(m)** | **MR5/Time (m)** | **Last Follow-Up Status** |
| --- | --- | --- | --- | --- | --- | --- | --- | --- | --- | --- |
| 45 | 8.6/F | TP53 p.E11Q 40.60%^#^ | - | - | NA | NA | Yes/NA | No/NA | No/NA | MMR |
| 49 | 8.4/M | KDM6A p.Q544* 4.30% | - | - | Yes | No | No/56 | No/56 | No/56 | MR2 |
| 52 | 6.1/M | ABL1 p.T315I 43.6% | ABL1 p.E255K 0.8% | - | No | No | Yes/14 | Yes/14 | Yes/14 | MR5 after HSCT |
| 56 | 6.5/F | ASXL1 p.G646Wfs*11 27.30% | - | - | Yes | Yes | Yes/3 | Yes/19 | Yes/19 | MR5 |
| 59 | 9.0/M | RUNX1 p.H105Q 8.20% | SETD1B p.D1090*fs*1 6.90% | - | NA | NA | NA | NA | NA | NA |
| 62 | 12.4/F | ASXL1p.G646Wfs*12 15.50% | - | - | No | No | Yes/29 | No/29 | No/29 | MMR |
| 64 | 6.1/F | ABL1 p.T315I 27.10% | ASXL1 p.E635Rfs*15 23.00% | ATG2Bp.L1041Ifs*19 5.90% | No | No | Yes/14 | Yes/14 | Yes/14 | MR5 after CBT |
| 67 | 11.4/M | KMT2A p.R2211Q 22.80% | - | - | Yes | Yes | Yes/9 | Yes/15 | Yes/15 | MR5 |
| 69 | 13.4/M | RUNX1 p.S314Cfs*285 45.90% | - | - | Yes | No | Yes/23 | No/23 | No/23 | MMR |
| 71 | 13.6/M | MAP3K7 41.80%^#^ | - | - | Yes | Yes | Yes/6 | Yes/15 | Yes/15 | MR5 |
| 72 | 12.3/F | PALB2 p.K569Rfs*8 47.60%^#^ | - | - | No | No | No/15 | No/15 | No/15 | MR2 |
| 74 | 8.8/M | ASXL2 47.40%^#^ | - | - | Yes | No | Yes/19 | No/19 | No/19 | MMR |
| 81 | 6.1/M | HLA-A p.R99Pfs* 24 8.80% | DDX41 48.30%^#^ | - | No | No | No/13 | No/13 | No/13 | MR1 |

HSCT, Hematopoietic Stem Cell Transplantation; CBT, Cord Blood Transplantation. #These variations were presumed to be of germline origin, but due to the lack of samples, it is impossible to be verify furtherly.
